# Sequential molecular analysis of circulating MCAM/MUC18 expression: a promising disease biomarker related to clinical outcome in melanoma

**DOI:** 10.1007/s00403-014-1473-7

**Published:** 2014-06-07

**Authors:** Maria Cristina Rapanotti, Tara Mayte Suarez Viguria, Gaetana Costanza, Ilaria Ricozzi, Andrea Pierantozzi, Alessandro Di Stefani, Elena Campione, Sergio Bernardini, Sergio Chimenti, Augusto Orlandi, Luca Bianchi

**Affiliations:** 1 Department of Laboratory Medicine, University of “Tor Vergata” Rome, Rome, Italy; 2 Department of Dermatology, University of “Tor Vergata” Rome, Policlinico di Tor Vergata, Viale Oxford, 81, 00133 Rome, Italy; 3 Department of Anatomic Pathology, University of “Tor Vergata” Rome, Rome, Italy

**Keywords:** Melanoma, MCAM/MUC18 expression, Disease biomarker, AJCC stages

## Abstract

MCAM/MUC18 is a cell adhesion molecule associated with higher incidence of relapse in melanoma. The purpose of our study was to evaluate its role as a promising disease biomarker of progression through sequential molecular MCAM/MUC18 RT-PCR assay on serial blood samples collected during the clinical follow-up of 175 melanoma patients in different American Joint Committee on Cancer (AJCC) stages. MCAM/MUC18 molecular detection, found at least once in 22 out of the 175 patients, was significantly associated with poor prognosis and death (*p* < 0.001), regardless of the AJCC stages. Positive expression, either if primarily present or later acquired, was associated with melanoma progression, whereas patients primarily negative or with subsequent loss gained clinical remission or stable disease, even if in advanced stages (*p* < 0.005). Six AJCC advanced stages always MCAM/MUC18 negative are in complete remission or with a stable disease (*p* < 0.007). Semiquantitative immunohistochemical MCAM/MUC18 staining on corresponding primary melanomas was related to peripheral molecular expression. Correlations between circulating molecular and tissutal immunohistochemical detection, primary tumour thickness, AJCC stages and clinical outcome were statistically evaluated using Student’s *t* test, ANOVA, Spearman’s rank correlation test, Pearson *χ* ^2^-test and McNemar’s test. In our investigation, MCAM/MUC18 expression behaves as a “molecular warning of progression” even in early AJCC patients otherwise in disease-free conditions. Achievement of this molecule predicted the emergence of a clinically apparent status, whereas absence or persistent loss was related to a stable disease or to a disease-free status. If confirmed in larger case series, MCAM/MUC18 molecular expression could predict good or poor clinical outcome, possibly becoming a promising prognostic factor.

## Introduction

MCAM/MUC18, a melanoma cell adhesion molecule, is recently obtaining more attention as a novel biomarker for disease progression and poor outcome in patients affected by melanoma [4, 38, 55]. Also cited as CD146, A32 antigen or S-Endo-1, it belongs to the immunoglobulin superfamily being primarily expressed at the intercellular junction of endothelial cells where it interacts directly with VEGFR-2 [26, 56, 58]. Originally identified in melanoma but not in normal tissue, it is now investigated in development, signal transduction, cell migration, mesenchymal stem cells differentiation, angiogenesis and immune response [60]. Many reports indicate that MCAM/MUC18 correlates with tumour thickness and metastatic potential of human melanoma cells in mice [20, 27, 29, 35, 62]. It is also an “ectopic” expression in primary cutaneous melanoma cells leading to increased tumour growth and metastasis in in vivo mouse models [18, 52]. If advanced and metastatic melanomas (80 %) strongly express MCAM/MUC18, the detection of this antigen on thin melanoma or benign melanocytic nevus is weaker and less frequent [18, 52]. In particular, SB-2 melanoma cell lines, commonly characterized by a low metastatic potential, do not regularly express MCAM/MUC18 but, when subsequently transfected with full-length human cDNA MCAM/MUC18 construct and injected in mice, easily develop metastases. As endothelial antigen, MCAM/MUC18 can affect angiogenesis-promoting neoplastic progression from local invasive to metastatic disease by up-regulating MMP-2 metalloproteinase and by cell interaction among extra-cellular matrix and vascular endothelial cells [63]. Mills et al. [37] studied the effect of a fully humanized anti-MCAM/MUC18 antibody (ABX-MA1) on tumour growth, angiogenesis and metastasis of human melanoma. ABX-MA1 treatment of melanoma cells was able to inhibit in vitro the promoter and collagenase activity of MMP-2, resulting in decreased invasion through Matrigel-coated filters. Reduced MMP-2 expression was also observed in implanted tumours in vivo [33, 37]. All these findings strongly support a reliable role of MCAM/MUC18 in melanoma progression. Several multiple marker RT-PCR assays have been demonstrated and proposed as sensitive methods to evidence circulating melanoma cells (CMCs) in peripheral blood of melanoma patients through the detection of one or more melanoma-associated markers (MAMs) of differentiation which include MCAM/MUC18 [2, 9, 10, 15, 21, 31, 34, 43, 44, 51, 53, 57]. Using a highly specific and sensitive multi-marker RT-PCR assay, we could document that among the five MAMs investigated (Tyr-OH, MART-1, MAGE-3, p97, MUC18/MCAM), only MCAM/MUC18 was statistically associated (*p* < 0.009) with advanced American Joint Committee on Cancer (AJCC) stages and with higher incidence of recurrences (95 % CI 2.9–374) [48, 49].

The purpose of this study was to extend our analysis to a larger series of patients exploring circulating MCAM/MUC18 expression by RT-PCR assay on serial blood samples obtained during the clinical course of the disease. Possible correlations among peripheral molecular monitoring and immunohistochemical MCAM/MUC18 staining on corresponding primary neoplasms, primary tumour thickness, AJCC stage and clinical outcome will be investigated in order to suggest additional tools of stratification and/or distinction for tumour progression. Statistical analyses will be performed to investigate the significance of MCAM/MUC18 expression between patients’ groups and controls.

## Methods

### Patients and healthy donors

One hundred and seventy-five melanoma patients entered prospectively this study. Information and consent forms, previously approved by ethical local Institutional Review Board (Code #2001068929_003, were provided at diagnosis, together with the permission to collect blood samples for research purposes. Patients were considered eligible if they had a histologically and immunohistochemically (S-100, HMB-45 and MART-1) confirmed diagnosis of malignant melanoma regardless of the time of the first diagnosis. Sentinel lymph node biopsies were performed in 15 (8.57 %) patients, showing a primary tumour thickness >1.0 mm or <1.0 mm if ulcerated or in T1B stage. All patients were treated at the Dermatology Department of the University of “*Tor Vergata*” Rome (Italy). According to the AJCC guidelines [3, 8], patients were classified as follows: seven patients (4 %) with in situ melanoma, 125 patients (72 %) in AJCC stage I, 29 patients (16.57 %) in AJCC stage II, six patients (3.42 %) in AJCC stage III and eight patients (4.57 %) in AJCC stage IV (Table 1). Two patients affected by metastatic disease secondary to occult primary melanoma were in AJCC stage III (UPN 2, UPN 25), while three were in AJCC stage IV (UPN7 UPN20, UPN28).

**Table 1:** Patients’ demographic and clinical characteristics

	No	%
Sex		
Female	86	–
Male	90	–
Age (years)	4.2 (mean)	27–72 (range)
Time from diagnosis (years)	0–21	
Median	1	–
Mean	1.78	–
AJCC stage^a^		
In situ	7	3.98
I	125	71.60
II	29	16.47
III	6	3.40
IV	8	4.54
Primary tumour site		
Head and neck	14	7.95
Trunk	91	51.70
Extremity	65	36.93
Unknown	5	3.40
Clinically evident disease	1	8.57
Clinically disease-free	1.64	91.4

^a^The AJCC staging was evaluated at the time of the blood draw after the diagnosis of primary melanomas or the diagnosis of first distant metastases in case of occult primary melanomas

The morphological and histological characteristics were as follows: seven in situ malignant melanomas, 113 thin malignant melanomas, 49 malignant melanomas, five occult melanomas and one uveal melanoma (Table 1). All metastatic patients were surgically treated, whenever possible.

One hundred and sixty patients (91.4 %) were considered clinically disease-free via conventional physical examination and imaging, while 15 (8.57 %) showed evidence of metastasis. Forty-six patients were checked only once, and the other 122 were serially sampled throughout the study and staged as follow: 90 patients in AJCC stage I, 21 in stage II, five in stage III and six in stage IV. We excluded the seven in situ malignant melanoma patients considering a molecular biomarker analysis performed in a population characterized by a very good prognosis as unnecessary.

Serial blood samples were collected from each patient starting from the date of the first melanoma diagnosis or the date of the first visit after the diagnosis of distant metastases (t0), and then every 6 months up to 3 years (median follow-up 20 months). Consequently, we established a molecular analysis of the follow-up collecting 122 samples at t1 (+6 months), 99 samples at t2 (+12 months), 30 at t3 (+18 months), 14 at t4 (+24 months) and 11 at t5 (+30 months). The date of the blood draw, obtained after the primary surgery or after the first diagnosis of distant metastases for occult melanomas (4/100), ranged from 0 to 20 years (median 1 year; mean 1.78 years; standard deviation 2,725; first quartile 0, third quartile 2) (Table 1). The AJCC staging was evaluated at the date of the first blood draw (Table 1). In addition to the RT-PCR MCAM/MUC18 assay, the patients were also checked for LDH, ALP, NSE and S100 peripheral blood levels. Blood samples from 50 healthy donors were taken from the Transfusion Centre as negative control population.

### Immunohistochemical studies

Histopathological diagnosis and post-surgical staging were routinely performed according to international criteria [3, 8, 41]. Immunohistochemical evaluation of MCAM/MUC18 expression was performed [5] in a subset of nineteen formalin-fixed and paraffin-embedded melanomas from selected patients (UPN3, UPN5, UPN6, UPN7, UPN9, UPN10, UPN11, UPN12, UPN13, UPN14, UPN15, UPN16, UPN17, UPN18, UPN19, UPN21, UPN23, UPN24) [5].

We analysed, as control population, 20 melanomas from our series, selected as they had never showed molecular MCAM/MUC18 expression and screened in line with matching for sex, age, ethnic background, primary tumour site and AJCC stages, as closely as possible. In this last series, we included six out of the 14 patients with evidence of disease (UPN23, UPN24, UPN25 in AJCC stage III and UPN26, UPN 27, UPN 28 in AJCC stage IV, respectively) who had never showed MCAM/MUC18 expression either at t0 or during molecular follow-up. After deparaffinization and blocking of endogenous peroxidase activity with 0.2 % H_2_O_2_ (20 min), immunostaining with rabbit polyclonal anti-MCAM/MUC18 (1:70, 1 h RT; Abcam, Cambridge, UK) was performed, followed by anti-rabbit IgG and amino-ethyl-carbazole (AEC) used as final chromogen. All procedures were performed at room temperature, using positive and negative controls. Semiquantitative MCAM/MUC18 immunohistochemical expression in melanoma cells was estimated at 200× magnification in at least ten fields [12] with an inter-observer variability <5 %, using a grading system in arbitrary units as follows: absent (0), low and focal (0.5) and positive (weakly positive 1+; moderately positive 2+; strongly positive 3+) staining intensity, as reported [13, 40, 42]. These scores were determined independently by two senior pathologists. For each case, we quantified the ratio of the total score with the number of analysed fields we had calculated.

### Cell lines

As positive control, we used the human melanoma cell lines M10 and M14, whereas the negative controls were represented by the breast cancer cell lines MCF-7 and MB-231 which do not express MCAM/MUC18 [15, 17, 56]. Cell lines were grown in RPMI-1640 (GIBCO-BRL) supplemented with 10 % foetal bovine serum (GIBCO-BRL) and antibiotics, in a humidified atmosphere with 5 % CO_2_ at 37 °C temperature. Cells were detached by trypsinization, then centrifuged, washed twice with phosphate-buffered saline (PBS) and stored at −70 °C, until use. M14 melanoma cells were serially diluted to mimic in vivo conditions of occult metastatic melanoma cells in blood and to establish sensitivity of our assay, starting from 1 × 10^6^ M14 cells mixed with 7 × 10^6^ cells from blood of healthy donors (BHD) up to one M14 melanoma cell as already described [49].

The specificity of the assay was checked using the two established melanoma cell lines M10 and M14. Neither MCAM/MUC18 mRNAs were detected when mRNA was isolated from breast carcinoma cell line (MB-231 and MCF-7), as documented [15, 17], nor MCAM/MUC18 transcripts were evidenced in the blood of our healthy donors [49]. To evaluate the level of detection, we performed serial dilutions of M14 melanoma cells in 6 ml blood from healthy donors, starting from 1 × 10^6^ M14 cells into 7 × 10^6^ cells from BHD, up to one M14 melanoma cell. After a single round of amplification (40 cycles), PCR products for MCAM/MUC18 were detected only when RNA was isolated from blood containing 100 or more melanoma cells, while the nested PCR brought the sensitivity down even in the presence of a single melanoma cell. Regarding M14 RNA serial dilutions, MCAM/MUC18 transcripts were detected in samples containing 1 ng M14 RNA, after first round of amplification, or less (1 pg) after nested PCR [49], documenting the high sensitivity of these assays.

### RNA isolation

Blood samples from melanoma patients and healthy donors (5 ml) were collected in PAX gene tubes (PreAnalytix–QIAGEN Hombrechtikon, CH) containing an additive for the collection of whole blood and a cellular RNA stabilizer. Samples were centrifuged (1,500*g* for 10 min), and the supernatant was discarded. The blood cells pellet was then frozen at −70 °C in a guanidine isothiocyanate solution. RNA from whole blood and from melanoma and carcinoma cell lines was extracted as described by Chomczynski and Sacchi [7], with slight modifications. RNA was further resuspended in distilled sterile water, and purity and amount were determined spectrophotometrically. Serial dilutions of M10 and M14 RNAs from 1 μg to 1 pg in 1 μg of healthy-donor RNA were also performed [49].

### RT-PCR methods

Two micrograms of total RNA and 2.5 U of Moloney murine leukaemia virus reverse transcriptase (Applied BioSystems, Roche Molecular Systems, Inc., Branchburg, NJ, USA) were used in all RT-PCR experiments, according to the manufacturer’s instructions. First-strand cDNA was generated with 2.5 μM oligo d(T)_16_, 5 mM MgCl_2_, 1 mM dNTPs and 1 U of RNase inhibitor (Applied BioSystems, Roche Molecular Systems, Inc., Branchburh, NJ, USA) during 1-h incubation at 42 °C. A 2-μl aliquot of cDNA was used for a multi-marker first-step PCR and a nested PCR. Primer sequences for MCAM/MUC18 were as described [9, 21, 49]. MCAM/MUC18 conditions for both first round and nested PCR were as follows: 94 °C for 1 min, 52 °C for 1 min and 72 °C for 1 min for 40 cycles. A hot start Taq was used in each amplification. The resulting nested products (25 μl) were analysed on a 2 % agarose gel. RNA integrity was checked electrophoretically, and the quality of cDNA was controlled by amplification of housekeeping genes such as β2-microglobulin or β-actin.

### Statistical analysis

Immunohistochemical results were analysed by means of Student’s *t* test and ANOVA. The differences were considered statistically significant for values of *p* < 0.05. Immunohistochemical expression was then correlated to the clinico-pathological features and to the MCAM/MUC18 peripheral *status* via Spearman’s rank correlation test. For statistical evaluations on detection of MCAM/MUC18 mRNA in peripheral blood of patients, due to the small size of our sample, we stratified the 175 melanoma samples into two different groups, early (AJCC stages I + II) and advanced (AJCC stages III + IV) AJCC stages. We collected 175 samples at t0, 128 samples at t1 (+6 months), 99 samples at t2 (+12 months), 30 at t3 (+18 months), 14 at t4 (+24 months) and 11 at t5 (+30 months). Time t4 and t5 were not considered due to the small number of samples. We used McNemar’s test to evaluate the accordance of the result (positive/negative) through time. The two patients groups, early and advanced, were associated with the clinical outcome (alive or dead). The association of the MCAM/MUC18 *status* with the follow-up was analysed via *χ* ^2^ test. Moreover, we distinguished and compared 14 samples in advanced AJCC stages III and IV in two categories: the former MCAM/MUC18 positive at t0 or later acquired during follow-up (eight patients) and the latter including patients who never showed MCAM/MUC18 (six patients). The differences were considered as statistically significant for values *p* < 0.05. SPSS 16 software program was used for statistical analysis.

## Results

### Patients’ characteristics

Patients’ demographic and clinical characteristics are shown in Table 1. One hundred-sixty-nine patients underwent primary surgery between 1987 and 2009; five patients were affected by metastatic disease from primary occult melanoma. One hundred-sixty-one patients out of the 175 patients were considered clinically disease-free at the date of the blood draw by conventional physical examination and imaging, whereas the remaining 14 showed evidence of the disease.

### MCAM/MUC18 immunohistochemical study

**Fig. 1:**
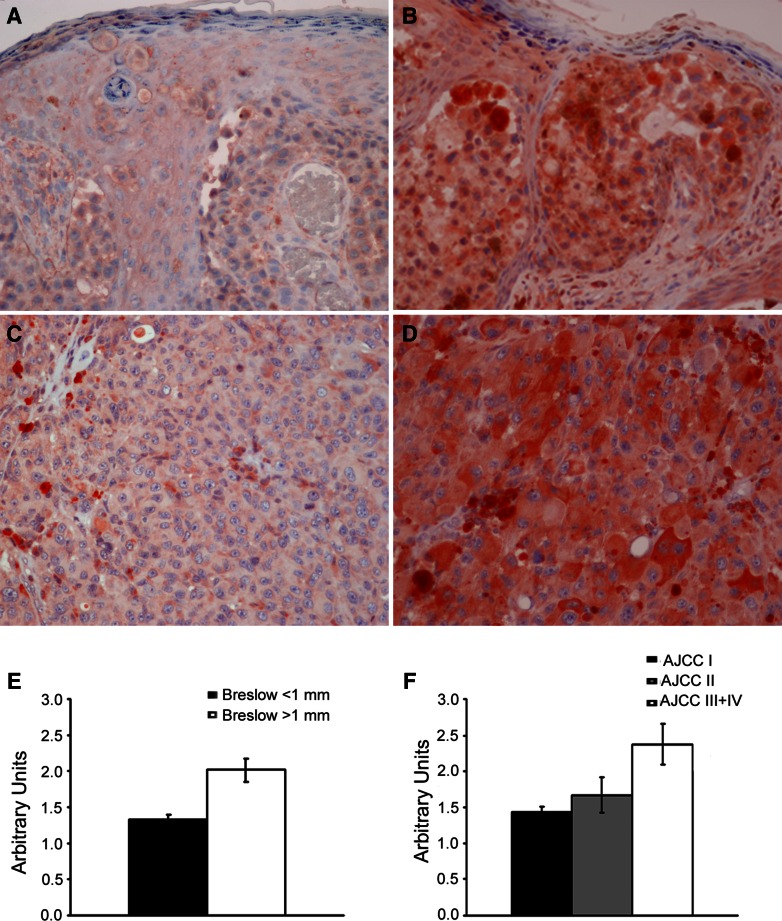
Immunohistochemical investigation revealed a constant and mainly cytoplasmatic positivity for MCAM/MUC18 antigen in melanoma cells (**a**–**d**). Semiquantitative analysis showed a variable result, with MCAM/MUC18 immunoreactions’ consistently increasing with both Breslow thickness and AJCC stages (*p* < 0.001 and *p* < 0.04, respectively) (**e**, **f**)

Immunohistochemical investigation revealed a constant and mainly cytoplasmatic positivity for MCAM/MUC18 antigen in melanoma cells (Fig. 1a–d). Semiquantitative analysis showed a variable result, with MCAM/MUC18 immunoreactions consistently increasing with both Breslow thickness and AJCC stages (*p* < 0.001 and *p* < 0.04, respectively) (Fig. 1e, f). Considering only thin melanomas (<1 mm thickness), one out of 14 cases was intensively MCAM/MUC18 positive (UPN13). As shown in Fig. 1, MCAM/MUC18 immunostaining intensity correlated positively with MCAM/MUC18 mRNA peripheral blood *status* (*p* < 0.001). Six III-IV AJCC stages patients (UPN23, UPN24, UPN25, AJCC stage III and UPN26, UPN 27, UPN 28, AJCC stage IV, respectively) who resulted always negative for MCAM/MUC18 expression either at t0 or during molecular follow-up were also negative for MCAM/MUC18 immunostaining. High statistical correlation between MCAM/MUC18 intensity and both Breslow thickness and AJCC stages was documented via Spearman’s rank correlation test. (*p* < 0.005 and *p* < 0.03, respectively).

### MCAM/MUC18 mRNA peripheral blood expression

We documented the presence of MCAM/MUC18 in the peripheral blood of 16 samples at t0 (9 %). Ten out of these 16 MCAM-/MUC18-positive patients (UPN4, UPN5, UPN6, UPN8, UPN9, UPN10, UPN11, UPN12, UPN13, UPN14) were classified in early AJCC stages (I–II), while six (UPN1, UPN2, UPN3, UPN7, UPN19, UPN20) in advanced AJCC stages (III–IV). In the complete early AJCC stages population, 111 patients could be serially sampled throughout the study and staged as follows: 90 patients in AJCC stage I and 21 in stage II. In detail, nine MCAM/MUC18 patients positive at time t0 (UPN4, UPN5, UPN8, UPN9, UPN10, UPN11, UPN12, UPN13, UPN14) permanently missed the biomarker expression. Only one patient (UPN6) was MCAM/MUC18 positive at t0, maintained the expression at t1 but later lost it at t2. Two patients in AJCC stage IA (UPN15 and UPN16) were firstly negative at t0 and then acquired a transient MCAM/MUC18 positivity at t1 which was subsequently lost at t2 and t3, respectively.

Patients UPN17 and UPN21, both in AJCC stage IIB, negative at t0 but afterwards acquiring MCAM/MUC18 expression within t1-t2, died before the t3 blood draw. The remaining ninety-seven out of 111 early-stage AJCC patients (78 in stage I and 19 in stage II) shared MCAM/MUC18 negativity in all molecular follow-up controls gaining on a good clinical outcome.

Considering the fourteen patients in advanced stages (UPN1, UPN2, UPN3, UPN7, UPN18, UPN19, UPN20, UPN22, UPN23, UPN24, UPN25, UPN26, UPN27, UPN28), six patients were MCAM/MUC18 positive at t0 (UPN1, UPN2, UPN3, UPN7, UPN19 and UPN20). Three out of these six did not even reach t1 since they died for disease progression (UPN1, UPN2, UPN3). Two out of the remaining three MCAM-/MUC18-positive patients maintained the expression until t3 and t4, but died afterwards (UPN19 and UPN20). Interestingly, the last MCAM/MUC18 positive at t0, a metastatic patient from primary occult melanoma (UPN7), after surgery and a vaccine therapy, missed definitely the molecular expression remaining negative and is still disease-free after 8 years from first diagnosis. Finally, two patients MCAM/MUC18 negative at t0, namely UPN22 (AJCC stage III) and UPN18 (AJCC stage IV), acquired MCAM/MUC18 expression afterwards within t1–t2 dying before t3.

Our results were all confirmed in triple distinct experiments, all provided of positive and negative cell controls.

Six out of the 14 advanced patients (UPN23, UPN24, UPN25 in AJCC stage III and UPN26, UPN27, UPN28 in AJCC stage IV, respectively) never showed MCAM/MUC18 expression, either at t0 or during molecular follow-up, and are still alive or with a stable disease.

**Fig. 2:**
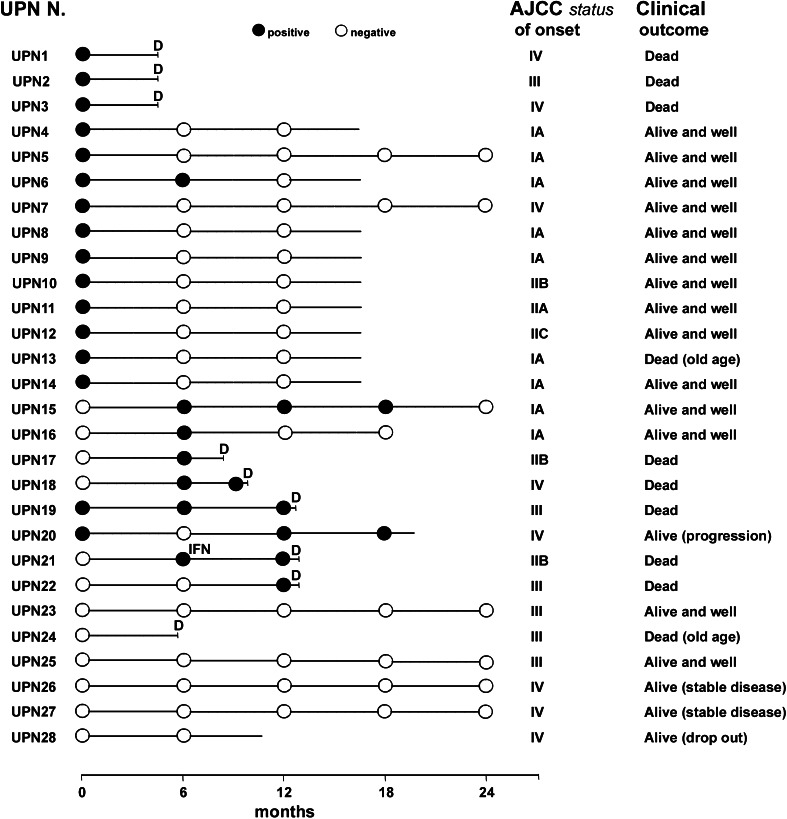
Peripheral blood MCAM/MUC18 mRNA expression analysed on each sample at onset and during follow-up, and clinical outcome of our series of patients

We documented that, by using MCAM/MUC18 RT-PCR assay, we could detect CMCs at all AJCC stages even long after surgical excision or treatment. Peripheral blood MCAM/MUC18 mRNA expressions, analysed on each sample at onset and during follow-up, and the clinical outcome of our series of patients, are described in Fig. 2.

### MCAM/MUC18 expression and statistical results

To correlate MCAM/MUC18 *status* (positive = 1 or negative = 0) and disease progression (alive or dead), we submitted to analyse only those patients for whom we could detect MCAM/MUC18 at least in two subsequent blood draws, starting from t0 up to the last follow-up draw, designing four classes based on the two *dummy variables* (alive/dead), as described in Table 2.

**Table 2:** MCAM/MUC18 expression detected at least in two subsequent blood draws (from t0 up to the last follow-up) is evaluated by designing two *dummy variables* alive/dead

MCAM/MUC18 classes	Disease progression (number of patients)		Total
	Alive	Dead	
0-0	2	0	2
0-1	0	4	4
1-0	11	0	11
1-1	0	2	2
Total	13	6	19

0-0: MCAM/MUC18 negative either at t0 or at the last follow-up draw

0-1: MCAM/MUC18 negative either at t0 but positive at the last follow-up draw

1-0: MCAM/MUC18 positive either at t0 but negative at the last follow-up draw

1-1: MCAM/MUC18 positive either at t0 or the last follow-up draw

MCAM/MUC18 expression detected at least in two subsequent blood draws (from t0 up to the last follow-up draw) is valuated by designing four classes based on the two *dummy* *variables alive/dead*

According to this model, we evidenced a strong association between MCAM/MUC18 *status* and clinical outcome. In particular, all patients belonging to the 0-1 class are dead, while all 1-0 class patients are alive and well (*p* < 0.001 and *p* < 0.005, respectively). Moreover, in the 14 patients in AJCC stages III–IV submitted to the same analysis, we documented a strong link between MCAM/MUC18 *status* (positive in eight patients and negative in six patients) and their clinical outcome: MCAM-/MUC18-positive patients died within few months (t2). Inversely, MCAM-/MUC18-negative patients are alive with stable disease or died of old age (two patients, UPN 13 and UPN 24) showing *p* < 0.007 value.

## Discussion

CMCs can be detected in a significant subset of patients with early-stage melanomas, and it has been shown that their circulating levels may have prognostic significance [28, 50, 54]. CMCs were firstly detected in melanoma patients by Smith et al. [57] and subsequently confirmed by several investigators [9, 10, 15, 21, 34, 43]. These cells are measurable in the peripheral blood either soon after the surgical excision of the primary tumours, regardless of their thickness, or in late-stage disease or even in clinically disease-free patients [14, 15]. These findings are confirmed by the percentage of positive cases for CMCs, ranging from 6 to 93 % of the reports [9, 10, 14, 19, 34, 53]. Multiple markers RT-PCR assay has been established as the most reliable and sensitive approach to identify CMCs in peripheral blood or in draining lymph nodes of melanoma patients [9, 10, 21, 22, 36, 39, 43, 44, 61], becoming a valuable potential technique for monitoring the disease *status* [16, 47, 54, 59]. We could document the high sensitivity of RT-PCR assay able to evidence MCAM/MUC18 up to a single CMC, in dilution experiments. Using this assay, we evaluated the co-expression of five MAMs, Tyr-OH, MART-1, MAGE-3, p97 and MCAM/MUC18, in melanoma patients stratified according to early and advanced stages of the disease. Previously, Pearl et al. [46] proposed to stratify MCAM-/MUC18-positive sentinel lymph node patients on the basis of melanoma cell adhesion molecule expression.

We demonstrated, by using a logistic regression univariate analysis, that MCAM/MUC18 level was a significant independent variable among patients with advanced disease [49]. More recently, Reid et al. [50] found that MCAM/MUC18 was significantly more common in non-surgically treated advanced melanoma patients with a negative treatment outcome than in those with a positive outcome (43 vs 9 %), reasonably related to an ineffective eradication of CMCs.

Our present investigations evidence a correspondence among MCAM/MUC18 mRNA blood level, detection and degree of expression of this marker on the corresponding primary melanoma tissue, tumour thickness, AJCC stages and clinical outcome. Our study shows that MCAM/MUC18 RT-PCR assay for CMCs correlates well with melanoma diagnosis and progression of the disease. Either if already detectable from the beginning or subsequently acquired during the course of the disease, MCAM/MUC18 is significantly associated with poor prognosis and death (*p* < 0.001). Differently from Reid et al. [50], we could detect MCAM/MUC18 positivity even in early AJCC stages (14 patients, ten at onset and four during follow-up), but surprisingly the patients who lost this marker are still clinically disease-free (12 out of 14). On the contrary, the two patients (AJCC stage IIB) who later acquired a persisting MCAM/MUC18 status sadly suffered from disease progression, dying before t3. The comparison of the clinical outcome of the twelve early AJCC stages patients, sharing fleeting expression with that of the two patients who later acquired a persisting expression, is significantly relevant (Table 2: *p* < 0.001 and *p* < 0.005, respectively). Thus, we believe that a sequential monitoring of MCAM/MUC18 *status*, even in a good prognosis population, may behave as a useful additional tool.

We designed four classes of patients to try to correlate MCAM/MUC18 *status* (positive/negative) and disease progression (alive/dead). Regardless of the AJCC stages, the absence, loss or positivity of this biomarker expression were associated with good or poor clinical outcomes, respectively. In particular, all patients belonging to the 0-1 class (MCAM/MUC18 later acquired) died, while all 1-0 class patients (MCAM/MUC18 lost at follow-up) are alive or in stable clinical condition (*p* < 0.001 and *p* < 0.005, respectively). To possibly explain the MCAM/MUC18 detection in twelve out of 175 patients in early AJCC stages, it is worthy to note that the peripheral blood draws were performed soon after the surgical removal of melanoma. This would be consistent with the proposal that MCAM/MUC18 is thought to play a role in cell–cell and cell–matrix interactions, being the surgical manipulation a possible cause of shedding of melanoma cells into circulation [23, 24, 32]. It is well known that not all the circulating tumour cells are able to colonize or metastasize since survival can be limited by immune surveillance or hemodynamic forces [25]. So, transient CMCs expressing MCAM/MUC18, either if related to the tumour burden or spread after the surgical excision, should be interpreted as limited survival of early micro-metastases with short half-life and consequent absence of clinical proliferating activity, while a persisting or later achieved MCAM/MUC18 detection could indicate a mature metastatic proliferative behaviour, able to extravasate into the surrounding tissue by degrading basement membrane and extracellular matrix [25, 30, 45].

Considering the patients in advanced stages, we emphasize a statistically significant difference if we compare the clinical course and outcome of those who were MCAM/MUC18 positive to those who never expressed this biomarker (*p* < 0.007). In particular, seven out of the advanced AJCC stages MCAM-/MUC18-positive patients suffered from disease progression and then subsequently died. The condition of the remaining patient is worth noting. The patient was at first diagnosed in advanced AJCC stage with MCAM-/MUC18-positive *status*, successfully achieved clinical remission after surgery and vaccine therapy, and then lost MCAM/MUC18 expression. He is now surprisingly disease-free being MCAM/MUC18 negative after 8 years from the first diagnosis of occult metastatic melanoma. Furthermore, the six advanced AJCC stages patients who never became MCAM/MUC18 positive are still alive with a stable disease.

Because of the high sensitivity of our method, capable to detect up to one melanoma cell diluted into 7 × 10^6^ healthy blood donor cells, we assure that this is a reliable tool useful to reproduce a minimal residual disease status in vitro. Thus, we are convinced that the lack of MCAM/MUC18 expression, as documented in the six clinically advanced patients, is a real biological *status.* These data are supported by the clinical course and outcome of these patients, as statistically reported in Table 2; moreover, when possible, molecular MCAM/MUC18 expression was correlated at t0 to the immunohistochemical negative staining on the primary tumours.

Recently, Capoluongo et al. [6] have reported an interesting commentary on the previous publication of Reid et al. [50] regarding the value of MCAM/MUC18 quantitative real time in clinical diagnostic. The authors underline that MCAM transcripts may fluctuate in a significant way in the healthy population. They suppose that the elevated copy number, also present in normal individuals, could be related to one of the two MCAM/MUC18 isoforms. Therefore, they hypothesize a possible MCAM transcripts overestimation mainly due to the short isoform widely expressed by endothelial cells, rather than the more melanoma-specific long isoform [1, 11]. By using the primers designed by Hoon et al. [21, 22], which map at the 5′upstream of the transcript (NM_006500-3,332 bp), and can select up to six putative isoforms, we cannot discriminate between the two previously mentioned isoforms. Despite this important impasse, we believe to have assessed a highly specific and sensitive test capable to detect the 436 bp (first PCR cycle) and the 262 bp transcripts (nested PCR) on melanoma peripheral blood samples and thus have achieved a statistically significant correlation between its positivity and clinical outcome. Moreover, in our experience, the detection of MCAM/MUC18 transcripts only in a small subset of patients—twenty-two out of 175 melanoma patients—does not fit the hypothesis of a possible overestimation, leading to regard our finding as an expression of a real biological status related to melanoma.

In our subset of melanoma tissues immunohistochemically tested, MCAM/MUC18 staining showed a consistent cytoplasmatic expression in melanoma cells interestingly related to an increased tumour burden, as observed on nude mice where a significant correlation between MCAM/MUC18 and metastatic growth was revealed [20, 27, 29, 35, 62]. We could also emphasize that MCAM/MUC18 proportionally increased with higher Breslow thickness and advanced AJCC stages, as observed by Pacifico et al. [42] and Reid et al. [50]. On the other hand, when possible, molecular MCAM/MUC18 expression was correlated to the immunohistochemical negative staining on the primary tumours. Effectively, molecular MCAM/MUC18 negativity documented in the six poor outcome melanoma patients out of the 14 AJCC III–IV stages was correlated with absence or low level of MCAM/MUC18 antigen staining. To the best of our knowledge, we documented for the first time a positive correspondence between MCAM/MUC18 immunostaining and mRNA MCAM/MUC18 peripheral blood expression.

Taken together, sequential molecular detection of MCAM/MUC18 seems to identify a subset of high-risk melanoma patients with poor prognosis. Since our data on the sequential MCAM/MUC18 expression were obtained from a relatively small cohort of melanoma patients, we cautiously chose the term “disease biomarker” to discuss its possible role in melanoma progression. Nevertheless, as the achievement of this molecular transcript, after being negative, predicted a clinically apparent disease, we hypothesize that the course of the MCAM/MUC18 *status* could be correlated with the clinical outcome, possibly becoming a prognostic marker. If the achievement of MCAM/MUC18 positivity is transitory, patients should not develop progression of melanoma disease; on the contrary, when persistent, patients could be at an increased risk of recurrence. In this case, MCAM/MUC18 positivity should be considered as a molecular predictor of recurrences, disease progression or risk of relapse.

MCAM/MUC18 could be also proposed as immunohistochemical marker of high-risk melanocytic lesions with metastatic potential. MCAM/MUC18 RT-PCR assay, even if not practical for routine melanoma diagnosis due to its low specificity, may improve the accuracy of staging and monitoring a specific subset of melanoma patients.

Our results, if confirmed in a larger series, could indicate that the course of MCAM/MUC18 expression could become a promising, independent prognostic marker in CMCs. Our experience highlights MCAM/MUC18 expression as a biomolecular warning of progression, not as a yet well-validated staging risk factor. We are aware that our results cannot lead now to a prompt change of the standard of care of patients with melanoma, but nevertheless they are valuable to be validated.
